# Building a Foundation for High-Quality Health Data: Multihospital Case Study in Belgium

**DOI:** 10.2196/60244

**Published:** 2024-12-20

**Authors:** Jens Declerck, Bert Vandenberk, Mieke Deschepper, Kirsten Colpaert, Lieselot Cool, Jens Goemaere, Mona Bové, Frank Staelens, Koen De Meester, Eva Verbeke, Elke Smits, Cami De Decker, Nicky Van Der Vekens, Elin Pauwels, Robert Vander Stichele, Dipak Kalra, Pascal Coorevits

**Affiliations:** 1 Department of Public Health and Primary Care, Unit of Medical Informatics and Statistics, Ghent University, Ghent, Belgium; 2 The European Institute for Innovation through Health Data, Ghent, Belgium; 3 Department of Cardiovascular Sciences, University Hospitals Leuven, Leuven, Belgium; 4 Data Science Institute, Ghent University Hospital, Ghent, Belgium; 5 Data Science, Algemeen Ziekenhuis Groeninge, Kortrijk, Belgium; 6 Department of Quality and Process Managenemt, Onze-Lieve-Vrouw Hospital, Aalst, Belgium; 7 Cell Business Intelligence, Algemeen Ziekenhuis Sint-Lucas, Ghent, Belgium; 8 Clinical Research Center, Antwerp University Hospital, Antwerp, Belgium; 9 University of Antwerp, Antwerp, Belgium; 10 Clinical Data Manager, General Hospital Maria Middelares, Ghent, Belgium; 11 Quality Department, General Hospital Maria Middelares, Ghent, Belgium; 12 Faculty of Medicine and Health Sciences, Heymans Institute of Pharmacology, Ghent, Belgium

**Keywords:** EHR, electronic health records, health data, data quality dimensions, data quality assessment, secondary use, data quality framework, fit for purpose, Belgium, data quality, framework, case study, hospital, variability

## Abstract

**Background:**

Data quality is fundamental to maintaining the trust and reliability of health data for both primary and secondary purposes. However, before the secondary use of health data, it is essential to assess the quality at the source and to develop systematic methods for the assessment of important data quality dimensions.

**Objective:**

This case study aims to offer a dual aim—to assess the data quality of height and weight measurements across 7 Belgian hospitals, focusing on the dimensions of completeness and consistency, and to outline the obstacles these hospitals face in sharing and improving data quality standards.

**Methods:**

Focusing on data quality dimensions completeness and consistency, this study examined height and weight data collected from 2021 to 2022 within 3 distinct departments—surgical, geriatrics, and pediatrics—in each of the 7 hospitals.

**Results:**

Variability was observed in the completeness scores for height across hospitals and departments, especially within surgical and geriatric wards. In contrast, weight data uniformly achieved high completeness scores. Notably, the consistency of height and weight data recording was uniformly high across all departments.

**Conclusions:**

A collective collaboration among Belgian hospitals, transcending network affiliations, was formed to conduct this data quality assessment. This study demonstrates the potential for improving data quality across health care organizations by sharing knowledge and good practices, establishing a foundation for future, similar research.

## Introduction

In an era of digital health care, hospitals are collecting structured and coded electronic health record (EHR) data at high velocity, resulting in a high volume of potentially valuable data [1,2]. The secondary use of these data specifically provides the opportunity to accelerate research and improve patient care pathways [3]. Belgium, already a major hub for clinical trials in Europe [4], is enhancing its reputation through the Real-World Data for Belgium [5] initiative, which collaborates with stakeholders to improve health care by reusing patient data for both primary and secondary purposes.

However, among these initiatives and opportunities that the evolving health care landscape presents, the quality of health data is imperative. Data quality is the cornerstone ensuring the trustworthiness and reliability of health data use and reuse. Despite its importance for safe patient-level care and accurate inferences, obtaining high-quality data in a health care setting remains a challenge, accompanied by ambiguities in defining data quality and the most suitable assessment methods [6-8].

A widely accepted definition in the literature is that of data being “fit for purpose” [9]. This concept is further refined by the Global Data Management Community (DAMA International), which adapts this definition to a more specific data context—“data quality is the degree to which the data dimensions meet requirements” [10]. This definition expands upon earlier ones by capturing the subjectivity and context-dependence inherent in data quality, offering a more stakeholder-sensitive perspective that aligns with the specificities of data use and reuse.

The path to ensuring data quality in the secondary use of health data is complex and multifaceted. It involves not only the original quality of the data when and where it is captured (eg, within the primary source such as an EHR system) but also the quality of the processes by which the data are transferred and transformed for further use, for example by mapping the data to a data model and terminology systems used within a clinical data warehouse. These stages, each embedded within the comprehensive data life cycle, are often overlooked in the literature [8], yet they are essential for a thorough understanding of data quality.

Prior to making secondary use of health data, it is crucial to measure the quality at the source and to establish methodologies for assessing relevant data quality dimensions. If not, it will not be possible to recheck the quality of the data at later life cycle points to verify that it has not been degraded (eg, through an extract, transformation, or load process). Research into data quality, especially when involving multiple primary data sources, consistently encounters significant challenges that add to the complexity of the research process [11]. These include measurement discrepancies [12], the use of varied software systems for data collection [13], inconsistent coding of diseases and procedures [12,14], and complex data sharing agreements [15]. Together, these factors not only hinder the efficient exchange of data but also significantly affect the quality of the data [16]. Furthermore, the health care ecosystem faces a significant challenge due to the lack of clear and practical guidelines for implementing strategies to ensure high data quality, especially when sharing data across different health care organizations for secondary use.

Several studies have aimed to define data quality dimensions and methodologies to describe and measure the dynamic complexity of data quality [6,17-20]. Despite these efforts, there is still no comprehensive framework that captures all aspects of data quality [8]. This has led to a fragmented understanding of data quality dimensions, with varying interpretations depending on the specific use of health data. Literature suggests that existing methods are often constrained by the absence of standardized metrics that can accurately assess data quality across different dimensions [8]. This limitation also extends to the transformation of these dimensions into concrete requirements for primary and secondary data usage, as well as for the extract, transformation, and load processes, that consider the original intent being the data’s collection at the primary source.

This paper examines these challenges by presenting a case study on data quality across multiple Belgian hospitals. The study is driven by a twofold objective—first, to evaluate the data quality of height and weight measurements across 7 Belgian hospitals, focusing on the dimensions of completeness and consistency; and second, to identify the challenges these hospitals encounter when implementing and managing data quality improvement initiatives.

## Methods

### Overview

In this case study the data quality framework developed by the European Institute for Innovation through Health Data (i~HD) was adopted [8,21]. This data quality framework is the prior result of analyzing 22 different published frameworks and consolidating all of their defined data quality dimensions into a consolidated framework, condensed into 9 distinct data quality dimensions. Table 1 presents the data quality dimensions incorporated into the i~HD data quality framework together with their definitions. As completeness and consistency dimensions were the most frequently used in the data quality literature, particularly when it comes to providing statistical assessment methods, these 2 were selected for quality assessment in this study [8,22].

**Table 1.** Data quality framework.

Data quality dimension	Definition
Completeness	The extent to which data are present
Consistency	The extent to which data satisfy constraints
Correctness	The extent to which data are true and unbiased
Timeliness	The extent to which data are promptly processed and up-to-date
Stability	The extent to which data are comparable among sources and over time
Contextualization	The extent to which data are annotated with acquisition context
Representativeness	The extent to which data are representative of intended use
Trustworthiness	The extent to which data can be trusted based on the owner’s reputation
Uniqueness	The extent to which data are not duplicated

### Case Study

#### Study Setting

The case study was conducted within 7 different hospitals across Belgium. Table S1 in Multimedia Appendix 1 presents a detailed table of the bed capacity for each participating hospital reflecting its scale and patient intake capability. To protect the confidentiality of the participating hospitals, they are referred to using numerical identifiers such as hospital 1, hospital 2, and so forth. This approach safeguards the privacy of all participating hospitals.

The variables of interest in this case study were height and weight. These 2 basic variables are both crucial health metrics that inform a wide range of clinical decisions [23-25]. These variables were used to exemplify the quality of data that should be reliably captured in all patient encounters, thereby serving as a barometer for the overall quality of data collection practices within a health care setting. To accomplish the objectives set in this study, we focused on 3 specific departments within each hospital, identified by their ward identifiers or department codes—C (surgical), G (geriatrics), and E (pediatrics). These ward identifiers are standardized across Belgian hospitals and refer to specific types of care provided within the hospital systems [26-28]. These codes are used for administrative purposes and ensure consistency in department identification across hospitals. However, they do not account for specific patient or disease characteristics, such as age or underlying health conditions. The use of ward identifiers provides a uniform method for extracting and comparing health data across similar specialty departments, allowing for standardized comparisons between hospitals in this study.

#### Data Collection

The data collection spanned for 2 years and included all patients discharged from the respective departments between January 1, 2021, and December 31, 2022. For every patient discharged within this time frame, the last recorded data for height and weight were extracted, which means that if multiple measurements were available, the most recent one was used. In cases where only a single measurement was taken, that value was used as the last recorded data. Should there be instances where measurements for these variables were not taken, these were systematically recorded as “NA” or “NULL” to denote the absence of data within the specified time frame. It is important to note that measurements taken during preadmission consultations were deliberately excluded from the data collection. This decision was made to ensure that the study focused on inpatient data, reflecting the data quality at this point of care during hospital stays. Since natural language processing falls outside the scope of this case study, only structured data were extracted. Table S2 in Multimedia Appendix 1 presents the total number of patients by department for each hospital involved in this case study.

The research team’s access to and analysis of the data were limited to aggregated results, for data protection reasons. Only hospital personnel with the appropriate access rights performed the data extraction and derived the data quality assessment results. The aggregated results were derived from the data quality assessments conducted within each hospital using a standardized set of analytical tools in R (version 2023/09/1; R Core Team) [29]. This approach not only facilitated the uniform assessment of data quality across various institutions but also eliminated the need for direct access by the research team to personal health information, thereby preserving patient anonymity and confidentiality within this research.

Data for this study were collected in accordance with data sharing agreements established with all participating hospitals (University of Leuven, University Hospital Gent, General Hospital Groeninge, OLV Hospital Aalst, General Hospital – AZ Sint-Lucas Gent, Antwerp University Hospital, and General Hospital Maria Middelares). The challenges and obstacles encountered by hospitals were highlighted during a conference session. During this session, various hospitals shared their experiences, and participants offered their insights and feedback [30-33]. All challenges were carefully documented and categorized into 3 main areas, that are privacy and governance requirements, software limitations, and institutional responsibilities. The categorization of obstacles was not predefined. Instead, it was developed through a consensus-based approach during the conference session. After gathering input from various hospitals, participants engaged in discussions and iterative feedback rounds until an agreement was reached on how to group the obstacles into these 3 categories. This process, culminating in a consensus among all participants, ensured that the identified obstacles were grouped and accurately reflected the shared experiences of participating hospitals.

#### Data Quality Assessment and Statistical Analysis

The first step in the data quality assessment focused on assessing the completeness of the collected data. Completeness, in this case study, refers to the extent to which height and weight data were recorded for patient admissions within the study’s time frame. Completeness was quantified as the percentage of missing values for both height and weight data across all selected departments.

The second step evaluated the consistency of the data. In this case study, consistency was determined by the percentage of recorded height and weight values falling within clinically acceptable ranges, predefined based on department-specific norms. To calculate consistency scores, missing data entries were excluded to avoid skewing the results. Subsequently, the proportion of data within the specified ranges for both height and weight was then determined, providing a percentage score of values falling inside and outside the acceptable range. Table 2 provides an overview of the variables and associated data quality rules for completeness and consistency.

**Table 2.** Overview of variables and data quality rules used for consistency.

Variable	Definition	Departments	Data quality rule
Height	Height (m)	Geriatrics (code G) Surgical (code C) Pediatrics (code E)	Code G: range between 1.4 and 2.2 meters Code C: range between 1.4 and 2.2 meters Code E: range between 0.4 and 2 meters
Weight	Weight (kg)	Geriatrics (code G) Surgical (code C) Pediatrics (code E)	Code G: range between 40 and 160 kg Code C: range between 40 and 160 kg Code E: range between 1 and 80 kg

### Ethical Considerations

The study protocol was approved by the Ethics Committee of Antwerp University Hospital (Project ID 6268).

## Results

### Data Quality Assessment

#### Data Quality Assessment for Completeness in Hospitals

**Figure 1.**
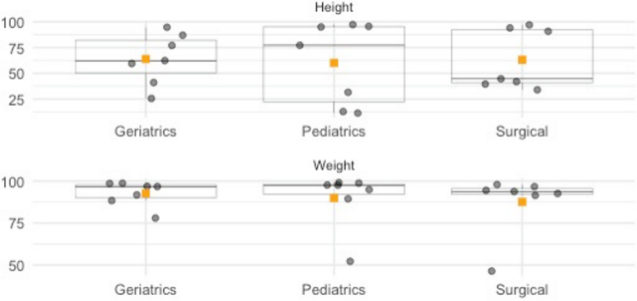
Completeness scores for height and weight data.

Figure 1 presents all completeness (%) scores for height and weight for each hospital across the described departments. Each box plot contains the IQR of the completeness percentage, with the central line within each box representing the median value. The square box presents the average value. Table S2 in Multimedia Appendix 1 compiles all results for each department within every hospital.

In the geriatrics department, the mean completeness of height data stood at 63.89%, with a median marginally lower at 62.35%. A wide range was observed, stretching from 25.56% to 94.70%, reflecting significant variability among institutions within this department, as evidenced by an IQR of 31.77%. Conversely, the pediatrics department presented a mean completeness of 60.11% with an even larger IQR of 73.10%, suggesting a more pronounced discrepancy in recording practices. Here, data completeness varied from a minimum of 11.29% to a maximum of 97.26%. The median value, at 77.24%, was higher than the mean, indicating a distribution skewed toward lower completeness percentages. The surgical department reported a mean completeness of 63.12% for height data, with an IQR of 51.75% underscoring the variability. The lowest recorded completeness was 33.92%, and the highest was 96.99%, with a notably lower median of 44.67% suggesting a skew toward less complete data.

A comparison with weight data showed different trends. The geriatrics department exhibited a high mean completeness of 92.73% and a relatively narrow IQR of 7.62% indicative of more uniform data collection practices. Completeness ranged from a high 77.97% to an exemplary 98.72%, with the median at 96.74%, pointing to a cluster of values toward the upper range. The pediatrics department’s mean completeness for weight data was 89.94%, with an IQR of 6.06%, denoting consistency. Despite some outliers, as the range spanned from 52.24% to 99.09%, the elevated median of 97.53% implied that most pediatric units adhered to high standards of data completeness. In the surgical department, the mean completeness was 87.63%, with a modest IQR of 3.56%, reflecting uniformity in data capture. With a range from 46.43% to 97.94% and a median of 93.79%, the findings suggested that while data recording is generally robust, there is room for improvement.

#### Data Quality Assessment for Consistency in Hospitals

**Figure 2.**
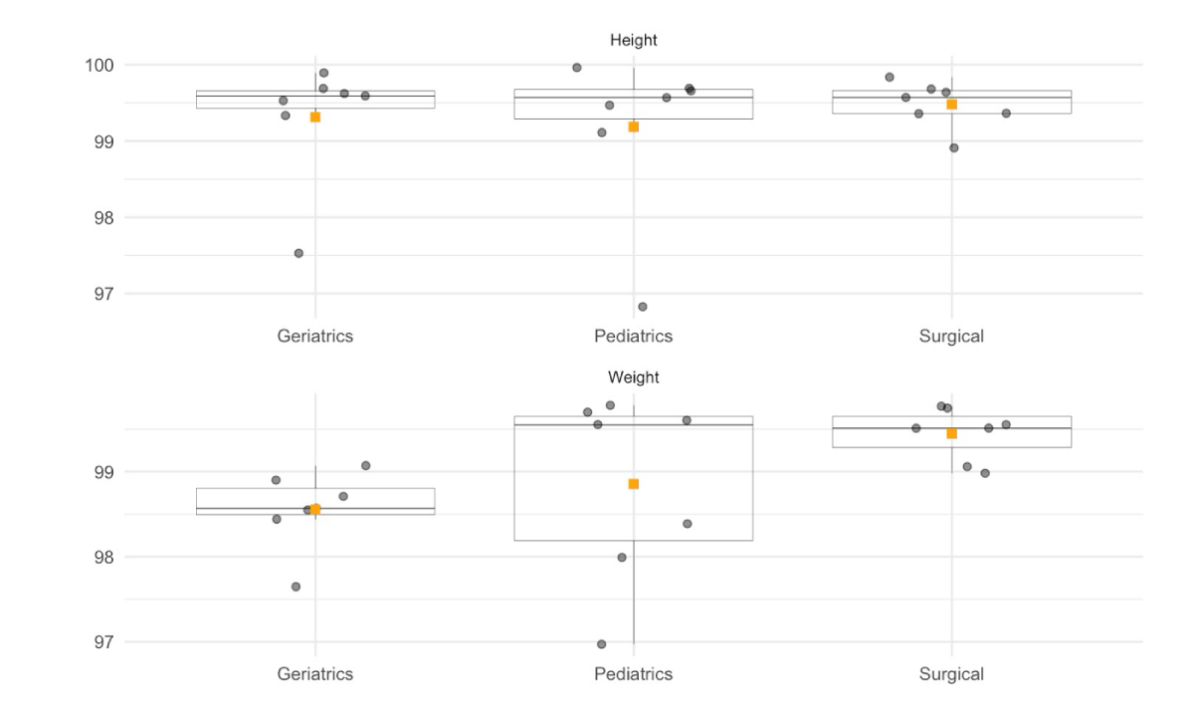
Consistency scores for height and weight data.

Figure 2 presents all consistency (%) scores for height and weight for each hospital across the described departments. Each box plot contains the IQR of the consistency percentage, with the central line within each box representing the median value. The square box presents the average value. Table S2 in Multimedia Appendix 1 compiles all results for each department within every hospital.

Height data recording demonstrated high consistency, with the geriatrics department achieving a mean of 99.31%. The consistency was impressively uniform, ranging narrowly from 97.53% to 99.89%, as the median at 99.59% and a minimal IQR of 0.23% confirmed. The pediatrics department’s mean consistency was 99.18%, with a slightly broader range from 96.83% to 99.96%. Nevertheless, a high median of 99.57% and an IQR of 0.39% indicated a strong overall consistency. Similarly, the surgical department showed a mean consistency of 99.48%, with a small range from 98.91% to 99.84%. The median of 99.57% and an IQR of 0.30% denoted a highly reliable level of data quality.

Consistency in weight data also exhibited positive results. In the geriatrics department, the mean consistency was 98.56%, with a tight range between 97.65% and 99.07%. The median mirrors the mean and an IQR of 0.31% highlighted the concentration of values. In pediatrics, the mean consistency was 98.85%, with a wider range from 96.97% to 99.78% and an IQR of 1.46%. Despite this variability, a high median of 99.55% was maintained. The surgical department continued the trend of high mean consistency at 99.45%, with a tight range and an IQR of 0.37%, signaling a consistent quality of weight data recording.

### Challenges and Obstacles Toward Data Quality Initiatives

#### Privacy or Governance Requirements

Data quality initiatives within hospitals are complicated by privacy and governance requirements that dictate the handling and sharing of patient data. These regulations are critical for protecting patient confidentiality but pose substantial challenges for collaborative data quality initiatives. An important challenge in this domain is the heterogeneity of documents concerning data sharing agreements. Each hospital, with its unique set of protocols and agreements, operates with its own distinct processes for data sharing. This fragmented approach toward data sharing and governance creates significant difficulties when attempting to share data across different hospitals. Not only does it impede the flow of information, but it also poses a barrier to improving data quality, as harmonizing data across different systems becomes a complex task.

#### Software Requirements

The diversity in hospital software systems, comprising both commercially available and internally developed solutions, poses a considerable challenge to initiatives aimed at ensuring data quality. Although we introduced a standardized protocol, the heterogeneity in these systems used by each participating hospital had an impact on the data extraction process. Additionally, while some hospitals extracted data directly from the original source, others retrieved it from their data warehouse. This variance in methods introduces multiple steps in the data extraction phase, each presenting areas for potential data quality issues even before the data quality assessments start.

Additionally, the variation in data management systems leads to differences in the data quality control protocols when entering data or there might not be designated structured fields for certain variables in the EHR system. Some participating hospitals indicated that although the completeness score was low, data for height and weight were available in the EHR. However, it was often embedded within unstructured fields, limiting its use for trend analysis and clinical decision support, and further complicating data harmonization efforts. All these differences in software tools used by the hospitals may lead to subtle yet impactful biases or variations in the data when data are transferred between hospitals.

#### Who Is Responsible?

An important challenge encountered in this study was the varying levels of responsibility for data quality across the hospitals. Collaboration with data scientists within each hospital was essential, as their expertise in handling health data was invaluable in acquiring the necessary information for the case study. Only 1 of the participating hospitals had a data quality manager responsible for data quality initiatives, and providing insights into existing issues, curation practices, and strategies for data quality improvement. However, it is important to note that there is no solid evidence showing that a hospital with a dedicated data quality manager is more likely to achieve higher data quality. The key seems to be a widespread organizational recognition of the importance of data quality, which can be upheld by either an individual or a team. This fragmented role of responsibilities also creates an ambiguity in formal education and training on data quality in the health care sector. The participating hospitals indicated they often resort to a “do-it-yourself” approach to tackle data quality, reflecting on an important dedication to obtaining high standards. Yet, this self-reliant method may lead to inconsistencies when data are shared with other entities, highlighting the need for a more uniform approach to data quality management and education.

## Discussion

### Principal Findings

The primary aim of this study was to assess the quality of height and weight data across 7 Belgian hospitals, focusing on the data quality dimensions of completeness and consistency. Our findings highlighted notable differences in the completeness of height and weight data across departments (eg, surgical, geriatric, and pediatric departments), with height data in the surgical and geriatric departments being particularly variable. This is likely due to the less frequent need to measure height compared to weight, which is more routinely measured given its critical role in patient care, such as medication dosage. In contrast, weight data showed consistently high completeness across all departments. Meanwhile, consistency scores for both height and weight were uniformly high, reflecting reliable data entry processes once measurements were taken. Our study and analysis align with other studies in suggesting that the quality of basic variables (eg, height and weight) may serve as a preliminary barometer for the overarching data quality within EHRs [7,34,35].

The mentioned departments were not selected at random but based on their distinct patient care characteristics and the importance of data quality related to their specialized care. Height and weight are important for clinical care decision-making in all 3 specialties. Surgery often involves immediate and precise measurements for both variables, for calculating anesthetic dosages and setting ventilation parameters during procedures [36]. In geriatric care, these measurements are crucial for monitoring nutritional status and adjusting medications, particularly given the frailty and complex health profiles of older patients [37]. In pediatrics, regular tracking of height and weight is vital for assessing growth, detecting developmental abnormalities, and ensuring accurate medication dosing to support overall health [38,39].

Additionally, the consistency ranges for these variables were set through consultations with the participating hospitals rather than from academic sources. This approach allowed us to tailor the acceptable ranges based on department-specific norms, ensuring that the assessment of consistency was relevant and practical within the specific clinical settings of each hospital. By focusing on consistency within predefined ranges, we were able to measure the reliability of the data once it had been entered into the system.

A key distinction of our study, compared to other research, is that we conducted this assessment across multiple hospitals, focusing not only on data quality results but also on the challenges and obstacles encountered during the process. This broader approach provided valuable insights into the variability of data quality practices across different health care settings.

To fully understand the outcomes of a data quality assessment, it is crucial to document the methodology and tools used. This documentation enhances the transparency and reproducibility of the assessment process. However, this alone is insufficient to determine whether data are truly fit for purpose. Understanding how data were initially collected and extracted from the system is equally important. The lack of detailed information on data extraction methods, combined with the variations in software requirements and data collection processes between the hospitals, plays a crucial role in understanding the quality of the data. These gaps in information may contribute to the observed variability in the completeness of height and weight data across different departments at various sites.

Comparative analyses with existing research reveal similar discrepancies in data capture and variations across multiple studies [40-42]. By not only focusing on the data quality results but also identifying the structural reasons for variability, we gained crucial insights into why certain inconsistencies persist in the data capture process.

While the establishment of clear standard operating procedures for data collection and extraction could help mitigate such inconsistencies, they represent only 1 aspect of a broader framework known as data quality maturity. Data quality maturity refers to an organization’s progression in managing its data practices, gradually refining these approaches and practices over time [43]. While many maturity models emphasize technical factors, such as the software used in EHR implementation and digitalization [44], a more comprehensive approach should also address people and processes. This includes education and awareness efforts to ensure that health care providers understand the significance of high-quality data and its impact on patient care and operational decisions. This broader perspective is necessary to achieve a higher level of data quality maturity, ensuring that data are reliable for both clinical decision-making and operation efficiency [45].

The long-term success of data quality initiatives depends on a more integrated strategy—one that balances the right tools, structured processes, and active involvement of staff.

A key aspect of this holistic approach is the establishment of a dedicated data quality manager or a specialized team. These roles are crucial for continuously monitoring data quality efforts, identifying gaps, and implementing corrective measures. By centralizing the responsibility for data quality, the team can enforce standards, streamline processes, and build a culture of accountability throughout the organization.

Detailed information from the participating hospitals, which provides further evidence of the positive outcomes, is available in Table S3 in Multimedia Appendix 1. These testimonials highlight the tangible impact of our research, demonstrating a shared commitment across institutions to continuously improve data quality and management practices. This collective effort underscores the growing recognition of the importance of high quality for enhancing patient care and operational efficiency.

### Limitations

This study selected 2 data quality dimensions—completeness and consistency (by range)—while acknowledging the value of the other dimensions within our established framework. The focus on these dimensions reflects their prevalent use within the data quality literature [23-25] and their relevance to clinical data assessment [36-39]. The selection of height and weight alone as variables for analysis cannot fully represent the complex and varied nature of patient records, which include critical elements such as vital signs, laboratory findings, and prescribed medications. Therefore, future studies should include a broader spectrum of quality dimensions and clinical variables. This expansion is necessary to gain a more comprehensive view of data quality, the issues that affect reaching good data quality and strategies to address the multifaceted nature of health data within EHRs.

Furthermore, the study’s reliance on aggregated data for the purpose of comparing hospitals introduces a limitation. Each hospital independently conducted its analysis and shared only the aggregated results, which constrained the potential for a comprehensive, cross-institutional evaluation of data quality at the individual patient level. This method limits the ability to identify specific data quality patterns that might only emerge through detailed, patient-level analysis.

Another limitation is the use of ward identifiers, which group patients based on administrative classifications rather than patient-specific or disease-related characteristics. This restricts the granularity of the analysis and may obscure important variations in data quality within different patient populations across the departments.

### Conclusions

This study has underscored the complexities and challenges inherent in assessing and assuring the quality of health data across multiple hospital settings. Our findings indicated a significant variance in the completeness of height and weight data among hospitals, underscoring the need for improved data capture and extraction protocols. The high consistency scores within recorded data attest to the precision of documentation when height and weight measurements are recorded. Performing a data quality assessment across different hospitals is a complex process in which multiple challenges need to be addressed before statistical analysis can even start. The challenges identified through this case study, particularly regarding privacy, governance, usage of different software systems, and responsibility for data quality, emphasize the need for more standardized operation procedures and specialized roles within the data quality management domain. The establishment of dedicated data quality managers and standardized education could bridge these gaps, enabling more effective and uniform data quality assessments and improvements.

## Supplementary material

10.2196/60244 Multimedia Appendix 1 Detailed data and analysis supporting the study findings.

## Abbreviations

EHR: electronic health record
i~HD: European Institute for Innovation through Health Data
