# Supplementary material for: Building a Foundation for High-Quality Health Data: Multihospital Case Study in Belgium
Source: JMIR Med Inform. 2024 Dec 20;12:e60244. doi: 10.2196/60244 (PMC11683741; doi:10.2196/60244)
Supplement: Multimedia Appendix 1 [file medinform-v12-e60244-s001.docx]

**Table S1**

| **Participating hospital** | **Accredited Beds (N) [28]** |
| --- | --- |
| University Hospital Leuven | 1908 |
| University Hospital Gent | 1049 |
| General Hospital Groeninge | 1044 |
| OLV Hospital Aalst | 842 |
| General Hospital – AZ Sint-Lucas Gent | 779 |
| Antwerp Universtiy Hospital | 593 |
| General Hospital Maria Middelares | 542 |

**Table S2**

| Hospital 1 | | | | | |
| --- | --- | --- | --- | --- | --- |
|  | # visits | Weight | | Height | |
|  |  | Completeness | Consistency | Completeness | Consistency |
| Surgical | 22634 | 96,71% | 99,77% | 33,92% | 99,64% |
| Paediatrics | 6109 | 98,77% | 99,70% | 95,58% | 99,69% |
| Geriatrics | 6615 | 98,72% | 99,07% | 59,47% | 99,62% |
| Hospital 2 | | | | | |
|  | # visits | Weight | | Height | |
|  |  | Completeness | Consistency | Completeness | Consistency |
| Surgical | 21495 | 97,94% | 99,75% | 41,86% | 99,84% |
| Paediatrics | 4970 | 99,09% | 99,55% | 97,26% | 99,96% |
| Geriatrics | 6662 | 98,57% | 98,90% | 41,07% | 99,89% |
| Hospital 3 | | | | | |
|  | # visits | Weight | | Height | |
|  |  | Completeness | Consistency | Completeness | Consistency |
| Surgical | 25880 | 46,43% | 98,98% | 39,52% | 98,91% |
| Paediatrics | 7797 | 52,24% | 97,99% | 31,54% | 96,83% |
| Geriatrics | 2531 | 88,42% | 98,57% | 25,56% | 99,69% |
| Hospital 4 | | | | | |
|  | # visits | Weight | | Height | |
|  |  | Completeness | Consistency | Completeness | Consistency |
| Surgical | 24784 | 91,46% | 99,55% | 90,82% | 99,68% |
| Paediatrics | 3482 | 89,43% | 99,78% | 12,84% | 99,11% |
| Geriatrics | 4475 | 77,97% | 98,71% | 77,07% | 99,33% |
| Hospital 5 | | | | | |
|  | # visits | Weight | | Height | |
|  |  | Completeness | Consistency | Completeness | Consistency |
| Surgical | 31961 | 92,59% | 99,51% | 44,67% | 99,36% |
| Paediatrics | 8356 | 94,89% | 98,39% | 77,24% | 99,66% |
| Geriatrics | 3485 | 91,79% | 98,44% | 62,35% | 99,59% |
| Hospital 6 | | | | | |
|  | # visits | Weight | | Height | |
|  |  | Completeness | Consistency | Completeness | Consistency |
| Surgical | 12905 | 93,79% | 99,51% | 96,99% | 99,57% |
| Paediatrics | 4090 | 97,53% | 99,60% | 11,29% | 99,57% |
| Geriatrics | 4391 | 96,74% | 97,65% | 87% | 99,53% |
| Hospital 7 | | | | | |
|  | # visits | Weight | | Height | |
|  |  | Completeness | Consistency | Completeness | Consistency |
| Surgical | 10179 | 94,46% | 99,07% | 94,07% | 99,36% |
| Paediatrics | 3611 | 97,66% | 96,97% | 94,99% | 99,47% |
| Geriatrics | 556 | 96,88% | 98,55% | 95% | 97,53% |

**Table S3**

| **Hospital** | **Quote** |
| --- | --- |
| General Hospital Groeninge | “These results demonstrate the critical urgency to draw attention to the importance of data quality within the hospitals. A thorough data quality analysis should be part of each real-world-data project conducted with or within the hospital.” |
| OLV Hospital Aalst | “Data quality starts with gaining support from healthcare professionals to enter the right data in the right place. “ |
| Antwerp University Hospital | “A standard uniform data sharing agreement (DSA) is needed. This would ensure that data exchange for similar projects would be less complicated. Regarding the data quality results, it seems useful to perform a second use case taking into account parameters other than just weight and height and using additional dimensions for the analysis. |
